# Structural Characteristics and Direct Liquefaction Performance of Macerals in Yili Coal from Xinjiang

**DOI:** 10.3390/molecules31020375

**Published:** 2026-01-21

**Authors:** Yongpan Wang, Xiaohui Li, Lang Liu, Xiaodong Zhou, Ting Liu, Guangjun Li, Jingxian Han, Yourlani Abudurgman

**Affiliations:** State Key Laboratory of Chemistry and Utilization of Carbon Based Energy Resources, College of Chemistry, Xinjiang University, Urumqi 830017, China; wangyongpan13@163.com (Y.W.);

**Keywords:** coal, macerals, screening method, structural characteristics, direct coal liquefaction, liquefaction oil

## Abstract

Effectively separating and utilizing macerals based on their properties is crucial for the efficient and high-value utilization of coal. This study enhances the traditional screening method by employing primary and stepwise crushing techniques to separate Yili coal (YLC) into inertinite-rich (YLI) and vitrinite-rich (YLV) concentrates. The structural characteristics and direct coal liquefaction (DCL) performance of YLC, YLV and YLI are subsequently studied. The results indicate that YLV exhibits the highest yield of oil, asphaltene and gas, a finding closely linked to its elevated content of highly active functional groups and its long aliphatic and bridge chains. Furthermore, the liquefaction oil from YLV contains the highest content of alkanes and phenols, which is attributed to its high content of aliphatic hydrocarbons and phenolic hydroxyl groups. In contrast, YLI exhibits the lowest product yield relative to YLC and YLV, with the highest contents of aromatics, esters, and ketones in its oil, due to its high contents of aromatic and carbonyl carbon. The separation, structural characteristics and DCL studies of macerals from Yili coal offer valuable insights for the efficient separation and utilization of macerals.

## 1. Introduction

Direct coal liquefaction (DCL) is a crucial technology for converting low-rank coal into liquid fuels and chemicals [1,2,3,4,5]. Its reaction conditions are relatively harsh, and the requirements for coal quality are also very strict. It is well known that coal is composed of three macerals: exinite, vitrinite and inertinite [6], which exhibit different liquefaction behaviors. The yields of their liquefaction oil follow the order of exinite > vitrinite > inertinite [7,8]. Therefore, the separation and utilization of macerals according to their properties can significantly enhance the efficient application of coal [9,10,11,12]. Macerals can be separated through hand picking, gravity separation, flotation and screening based on their physicochemical differences. Hand picking is a visual method that distinguishes and separates macerals based on macroscopic characteristics, such as color and luster. Although this method is user-friendly and retains the chemical properties of the macerals, it suffers from low efficiency and high subjectivity, making it suitable only for coal with prominent macroscopic characteristics, and it is not conducive to batch production [13]. In contrast, gravity separation is a method that separates macerals based on their density differences and is suitable for industrialization. However, this method exhibits strong dependence on water resources and requires efficient drying equipment to treat the products, which greatly increases costs [14]. Flotation separation is a process that separates macerals based on differences in their interfacial properties and hydrophobicity [15]. Typically, the differences in surface properties among macerals are minor, making it challenging for flotation to achieve highly selective separation of macerals [11,15,16]. Screening is a dry separation method that leverages the differences in crispness and hardness of macerals. The screening method is operationally simple and preserves the chemical properties of coal macerals. However, due to certain process limitations, the content of individual macerals in the products is typically less than 70% [16,17]. Therefore, developing a simple screening process to enrich a single maceral to over 80% is of great significance for studying the structural characteristics and transformation properties of different macerals.

Studying the structure of macerals is a practical approach to analyzing their differences in DCL performance and evaluating the yield and quality of liquefied oils [18,19]. Furthermore, the correlation between the liquefaction behavior and structural characteristics of macerals can provide theoretical support for developing more efficient and low-consumption DCL processes. For example, Shu et al. [20] established molecular structure models for vitrinite and inertinite from Shenhua coal. Based on bond-level parameters of the molecular model, they found that the liquefaction of vitrinite is thermodynamically controlled, whereas that of inertinite is dynamically controlled. By leveraging these distinct features, an efficient liquefaction technology for Shenhua coal was subsequently developed. Yu et al. [7] obtained exinite-rich and vitrinite-rich coal through isodensity gradient centrifugation. By combining ^13^C NMR, FT-IR and elemental analysis, the covalent bond concentrations in macerals were calculated. The DCL study revealed that the interaction between macerals promoted coal conversion and reduced hydrogen consumption. Lian et al. [21] developed models of the macerals from Heidaigou coal based on ^13^C NMR and XPS. They found that the significant difference in DCL performance between vitrinite and inertinite can be attributed to the higher content of aliphatic structures in vitrinite, whereas that of aromatic structures is higher in inertinite. These studies are crucial for establishing the relationship between structure and DCL of macerals, as well as their impact on the efficient conversion and utilization of coal. However, few studies have focused on the correlation between maceral structure and the liquefaction oil; therefore, this study aims to supplement this aspect.

This study explores the relationship between the crushing times and the maceral content of Yili coal. Utilizing an improved traditional screening method, YLI with an inertinite content of 83.08% is obtained through primary crushing and screening, while YLV with a vitrinite content of 80.80% is obtained through a stepwise crushing and screening process. Compared with the traditional screening method, this improved method increases the content of individual macerals. Additionally, compared to isodensity gradient centrifugation and flotation methods, this method is simpler and faster and requires no chemical reagents during separation. The distribution of functional groups and carbon structural characteristics of YLC, YLV and YLI are analyzed using FT-IR, Raman and ^13^C NMR spectra. The relationship between the structural characteristics and DCL behaviors of YLC, YLV and YLI is also investigated by autoclave experiments. Further analysis of the liquefaction oils reveals a strong correlation between the structure of each maceral and the composition of its corresponding liquefaction oil. This study provides theoretical and technical references for the green separation and utilization of macerals.

## 2. Materials and Methods

### 2.1. Preparation of Coal Samples

#### 2.1.1. Preparation of Raw Coal

The Yili coal (Xinjiang, China), which is classified as long-flame coal, was used in this study. The raw coal was crushed by a crusher (SDHD 150t, Sundy, Changsha, China) and then dried in a vacuum oven at 80 °C for 12 h. The dried coal was subsequently ground to a particle size of <0.045 mm (designated as YLC). The maceral composition of YLC is analyzed by microscopy (MSS-2000, Ruike Technology Co., Ltd., Beijing, China) and is presented in Table 1. The mineral components were disregarded in the subsequent research due to their low content.

#### 2.1.2. Separation of Inertinite by the Primary Crushing Method

The dried coal (Section 2.1.1) was screened according to the GB/T19093-2003 [22]. The maceral composition of coal with different particle sizes is presented in Table 2. As shown in Table 2, with particle size decreasing, the content of inertinite rises while the content of vitrinite and exinite diminishes. The content of inertinite reaches 83.08% in the coal with a particle size < 0.045 mm, which is designated as YLI.

#### 2.1.3. Separation of Vitrinite by the Stepwise Crushing Method

The dried coal (Section 2.1.1) was ground by a grinder (SDPP 1002, Sundy, Changsha, China) in six steps, each for 10 s. The coal with a particle size > 0.074 mm was subjected to further grinding. The maceral composition of coal with different grinding times is detailed in Table 3. With the number of grinding times increased, the content of vitrinite and exinite increased progressively, while the content of inertinite decreased. After the fifth grinding, the coal sample achieves a particle size of <0.074 mm. Subsequently, the sample was sieved using a 0.045 mm mesh, revealing a vitrinite content of 80.80% in the fraction > 0.045 mm. To minimize the influence of particle size on DCL, the coal fraction > 0.045 mm obtained after the fifth grinding was subjected to a sixth grinding to <0.045 mm and then designated as YLV. YLC, YLI and YLV were desiccated in a vacuum oven at 80 °C for 24 h to minimize the influence of the moisture. The proximate and ultimate analyses of YLC, YLV and YLI are shown in Table 4.

### 2.2. Direct Coal Liquefaction Experiment

Experiments were conducted in a 100 mL autoclave (Tongda Reactor Factory, Dalian, China). YLC (daf, 6 g) and tetralin (THN, 24 g, Macklin, Shanghai, China) were added to the autoclave. The autoclave was purged with H_2_ three times and then pressurized to 7 MPa with H_2_. The autoclave was heated to 430 °C and maintained at this temperature for 40 min; it was then cooled to room temperature. The DCL experiments of YLI and YLV followed the same procedure as that of YLC. The gaseous products were collected and tested by gas chromatography (GC-2014C, SHIMADZU, Nakagyo-ku, Japan). The liquid–solid mixture was extracted using a Soxhlet apparatus with hexane (INBOTE, Tianjin, China) and tetrahydrofuran (INBOTE, Tianjin, China). Yields of oil product and asphaltene were calculated based on the initial mass of THN, coal ash, moisture content, the hexane soluble fractions and tetrahydrofuran soluble fractions. The calculation formulas are as follows:

(1) $$Y_{Re}\;=\;\frac{m_{\left(Re\right)}\;-\;m_{\left(Cat\right)}}{m_{\left(daf\right)}}\;\times 100\%$$

(2) $$Y_{AP}=\frac{m_{\left(AP\right)}}{m_{\left(daf\right)}}\times 100\%$$

(3) $$Y_{\mathrm{Gas}}=\frac{\left(P_{0}+P_{2}\right)\times \left(V_{0}-V_{1}\right)\times T_{0}}{P_{0}\times {\;T}_{2}\times {\;V}_{m}\times {\;m}_{\left(daf\right)}}\times \sum_{i}\left(\frac{R_{i}U_{i}}{100}\right)\times 100\%$$

(4) $$Y_{Oil}=1-Y_{Re}-Y_{AP}-Y_{\mathrm{Gas}}$$

## 3. Results

### 3.1. Structural Characteristics of Macerals from Yili Coal

#### 3.1.1. FT-IR Spectrum

**Figure 1:**
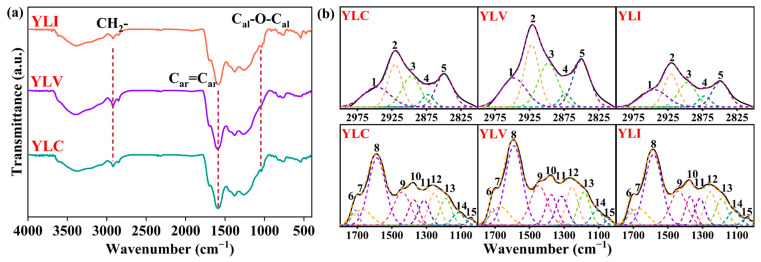
(**a**) FT-IR spectra of YLC, YLV and YLI; (**b**) the FT-IR fitting curves of YLC, YLV and YLI.

It is important to study the functional groups of different macerals to evaluate their properties in DCL [23]. Figure 1a illustrates the FT-IR (BRUKER VERTEX70, Bremen, Germany) spectra of YLC, YLV and YLI. As shown in Figure 1a, the peak at 2921 cm^−1^ associated with CH_2_- asymmetric stretching vibration in YLV exhibits the highest intensity. Conversely, the most prominent peak for YLI is the C-O-C stretching vibration peak of alkyl ether at 1039 cm^−1^. This indicates that YLV contains a greater abundance of aliphatic structures, while YLI is richer in alkyl ether structures. Then, the peaks at 3000–2800 cm^−1^ and 1800–1000 cm^−1^ [24,25,26,27] are fitted as shown in Figure 1b (OriginPro 2024b). Peak parameters are presented in Table 5. As shown in Table 5, the content of alcohol C-O, phenol C-O, CH_2_ and CH_3_ groups in YLV is the highest, while the content of these highly reactive functional groups in YLC and YLI is lower, which means the low reactivity of YLC and YLI in DCL. Additionally, the data in Table 5 indicate that the YLI exhibits the highest relative content of alkyl ether C-O-C, aromatic ether C-O, aromatic C=C, conjugated C=O and carboxyl C=O functional groups. The high content of aromatic C=C functional groups suggests that YLI contains more stable aromatic rings. A semi-quantitative analysis of aliphatic and aromatic functional groups is presented in Table 6. It reveals that YLV exhibits the highest content of aliphatic hydrocarbons, the longest aliphatic chains, and the highest aliphatic/aromatic ratio, indicating that more aliphatic and alkyl side chain structures are attached to the aromatic rings of YLV. Such structures are easily cleaved to generate radical fragments during DCL, which react with hydrogen donors to produce liquefied oil.

#### 3.1.2. Raman Spectrum

**Figure 2:**
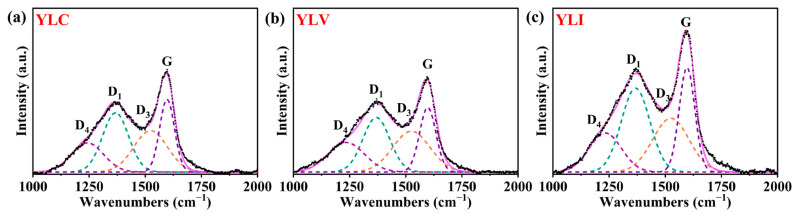
(**a**) Raman peak fitting diagrams of YLC; (**b**) Raman peak fitting diagrams of YLV; (**c**) Raman peak fitting diagrams of YLI.

As shown in Figure 2a–c, the defect peak (D peak) and the graphite peak (G peak) in YLC, YLV and YLI are displayed (Xplora Plus, HORIBA, Saint-Aubin-lès-Elbeuf, France), respectively. The D_1_ peak at 1368 cm^−1^ corresponds to the aromatic compounds with at least six rings [28], while the D_3_ peak at 1525 cm^−1^ relates to the amorphous structure of the mixed hybrids (sp^2^ or sp^3^) [29,30]. The D_4_ peak at 1237 cm^−1^ is associated with C-C stretching vibrations of aliphatic [31], and the G peak at 1597 cm^−1^ is caused by C=C bond vibration [32,33]. Table 7 details the D and G peak characteristics for YLC, YLV and YLI (OriginPro 2024b). Notably, YLV demonstrates the highest values for D_3_/G and D_4_/G, indicating a greater presence of amorphous structures and aliphatic carbon, which is consistent with the FT-IR results. Additionally, YLI exhibits the highest D_1_/G and lowest D_all_/G radio, indicating the greatest degree of aromatic condensation, and contains more stable ordered aromatic rings.

#### 3.1.3. ^13^C NMR Spectrum

**Figure 3:**
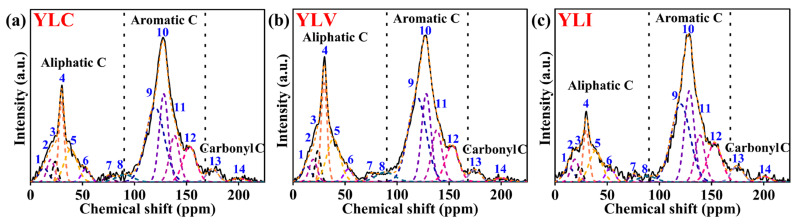
(**a**) ^13^C NMR peak fitting diagrams of YLC; (**b**) ^13^C NMR peak fitting diagrams of YLV; (**c**) ^13^C NMR peak fitting diagrams of YLI.

The ^13^C NMR (600 MHz Bruker Avance III, Switzerland) analysis was conducted to investigate the carbon skeleton of YLC, YLV and YLI. The fitted curves for the ^13^C NMR spectrum of YLC, YLV and YLI are shown in Figure 3a–c (OriginPro 2024b), respectively. The fitting results are detailed in Table 8. Chemical shifts for aliphatic, aromatic and carbonyl carbons are 0–90 ppm, 90–165 ppm and 165–225 ppm, respectively [34,35,36,37,38]. It reveals that aromatic carbon predominates in the three coal samples (90–165 ppm). Meanwhile, YLV exhibits the highest content of aliphatic carbon, YLI exhibits the highest content of aromatic carbon and carbonyl carbon. A semi-quantitative analysis of the structural parameters for the coal samples was conducted by consolidating the data from Table 8, and the results are presented in Table 9. As shown in Table 9, the C_n_ and C_b_ values of YLV are the highest, indicating that the average lengths of the methylene aliphatic and bridge chains of YLV are the longest. Additionally, the σ_-C_ value for YLV is the highest, but the σ_-O_ value is the lowest. Compared to YLC and YLI, the more aromatic carbon is substituted by carbon in YLV, indicating that more aliphatic chain structures are on the aromatic rings of YLV, which is consistent with the FT-IR results. The X_b_ values for naphthalene and anthracene are 0.20 and 0.29 [39,40], respectively. The X_b_ values of YLC, YLV and YLI are 0.25, 0.24 and 0.26, implying that the aromatic clusters in the three coal samples consist of 2–3 aromatic rings, and the aromatic clusters of YLI are the largest.

### 3.2. Direct Coal Liquefaction

#### 3.2.1. Results of DCL

**Figure 4:**
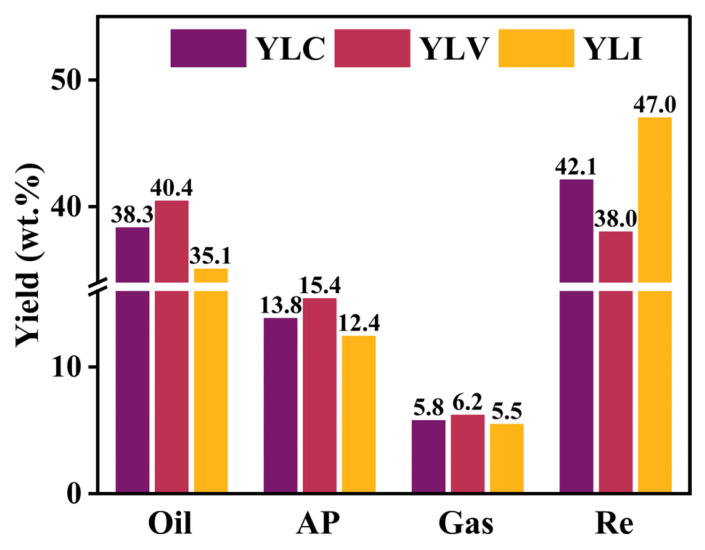
Results of DCL for YLC, YLV and YLI.

Figure 4 illustrates the results of DCL for YLC, YLV and YLI, comprising the yield of liquefied oil, asphaltene, gas and residue. The yields of oil, asphaltene and gas for YLV are 40.4, 15.4 and 6.2 wt.%, respectively, which are higher than those for YLC and YLI. Conversely, the residue yield of YLI is significantly higher than YLC and YLV, reaching 47.0 wt.%.

During DCL, the highly reactive groups, such as alcohol C-O and aliphatic CH_2_, CH_3_ groups, preferentially decompose from the carbon skeleton to form free radicals. These radicals then combine with active hydrogen and low-molecular-weight radicals to produce liquefied oil and gas. In contrast, the stable functional groups, such as aromatic ether C-O, aromatic C=C and conjugated C=O resist free radical generation during DCL. Long aliphatic and bridge chains are easily cleaved to generate radical fragments during DCL, which react with hydrogen donors to produce liquefied oil. Moreover, the high-molecular-weight free radicals interact to form higher-molecular-weight residues, resulting in the higher liquefaction residue rate for coal.

#### 3.2.2. Analysis of Liquefied Oil

**Figure 5:**
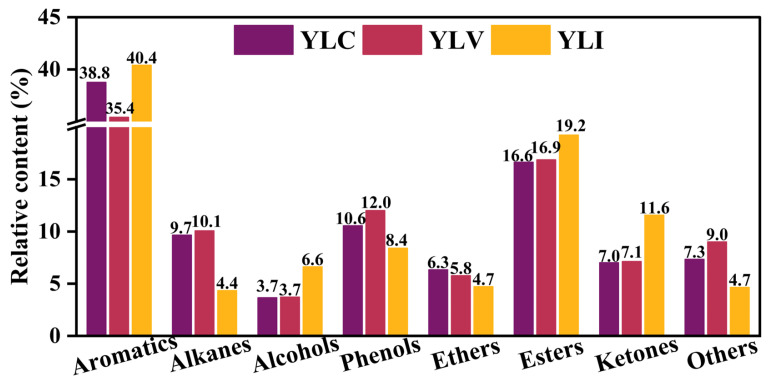
Liquefied oil composition for YLC, YLV and YLI.

The liquefied oils of YLC (Oil_YLC_), YLV (Oil_YLV_) and YLI (Oil_YLI_) are classified into eight groups based on the group composition by GC-MS (Agilent, The United States) [41,42]. As shown in Figure 5, the composition distribution of Oil_YLC_, Oil_YLV_ and Oil_YLI_ differs significantly; the content of aromatics in Oil_YLI_ is higher compared to Oil_YLC_ and Oil_YLV_. Conversely, the content of alkanes in the Oil_YLV_ is higher than in Oil_YLC_ and Oil_YLI_. Oil_YLI_ contains the highest content of esters and ketones, while Oil_YLV_ contains the highest content of phenols. Based on the analysis of FT-IR, Raman and ^13^C NMR, these distinct differences in the composition distribution of liquefied oil may correlate with the structural properties of YLC, YLV and YLI.

## 4. Discussion

The structures of the three coals are characterized using FT-IR, Raman and ^13^C NMR. The data show that YLV exhibits the highest content of highly reactive functional groups and aliphatic hydrocarbons, as well as the longest aliphatic and bridge chains. In contrast, YLI exhibits the highest content of aromatic carbon and the highest degree of aromatic condensation. The highly reactive functional groups preferentially decompose from the carbon skeleton to form free radicals, and long aliphatic and bridge chains are easily cleaved to generate additional free radicals. These free radicals then combine with hydrogen radicals to form liquefied oil. Therefore, the reactivity of the three coal samples follows YLV > YLC > YLI, and YLV generates free radicals more readily than YLC and YLI during DCL, resulting in the highest yields of oil, asphaltene and gas for YLV. Moreover, the high-molecular-weight free radicals are easily formed because YLI contains the largest aromatic cluster. These free radicals further interact to form higher-molecular-weight residues, resulting in the highest liquefaction residue rate for YLI.

Combined FT-IR and ^13^C NMR analyses indicate that YLI exhibits the highest content of aromatic carbon, resulting in a higher content of aromatics in the Oil_YLI_ compared to Oil_YLC_ and Oil_YLV_. Conversely, the content of alkanes in the Oil_YLV_ is higher than in Oil_YLC_ and Oil_YLI_, which is attributed to the highest content of aliphatic hydrocarbon and the longest aliphatic chain in YLV. Additionally, YLI contains higher contents of carbonyl groups and carbonyl carbon compared to YLC and YLV. In contrast, the YLV contains more phenolic hydroxyl groups compared to YLC and YLI. Consequently, Oil_YLI_ exhibits the highest content of esters and ketones, while Oil_YLV_ exhibits the highest content of phenols. The C-O bonds of alkyl ethers in coal are cleaved to form alcohols during DCL. The content of the alkyl ether group in YLI is higher compared to YLC and YLV, leading to a higher content of alcohols in the Oil_YLI_ than Oil_YLC_ and Oil_YLV_. These results indicate that the structures of macerals affect the composition distribution of their liquefied oils.

The traditional screening method features simple operation and low restrictions on coal types [16]; however, due to the low content of individual macerals in the separation products, it is often used for the rough separation of coal. Nevertheless, by controlling the particle size and number of crushing cycles, the primary and stepwise crushing methods can increase the content of individual macerals. The advantage of isopycnic gradient centrifugation lies in its ability to achieve high-precision separation, with the content of a single maceral in the product capable of exceeding 90% [34]. Flotation can enable the large-scale separation of coal macerals [11]. However, a commonality between isopycnic gradient centrifugation and flotation is the requirement for water and chemical reagents, whereas the primary and stepwise crushing method does not. Meanwhile, the correlation between the composition of liquefied oil and the structure of macerals is explained, providing a reference for the separation and utilization of macerals based on structural characteristics.

Techno-Economic Analysis (TEA) is a method for assessing the economic feasibility of technology, focusing on costs and benefits. Life Cycle Assessment (LCA) is a method for assessing the environmental sustainability of technology, covering the entire process from resource extraction to disposal. The separation of macerals incurs additional equipment investment and operational costs, which can be quantitatively evaluated by TEA. Meanwhile, TEA can also evaluate the economic benefits derived from the utilization of separated macerals. Furthermore, TEA can determine the most economically efficient integrated process pathway by modeling and calculating the total investment, operational costs and product value under different scenarios, providing economic theoretical support for the industrialization of macerals separation and utilization. LCA can quantitatively and systematically evaluate factors such as the carbon emissions, energy consumption and environmental impact associated with maceral separation and utilization, thereby revealing the optimal environmental management strategy. The data obtained in this study, including the maceral content, oil yield, conversion rate and liquefied oil composition of YLV and YLI, can serve as partial data for TEA and LCA of the maceral separation and direct liquefaction processes. LCA and TEA serve as critical bridges linking fundamental research to industrial applications, enabling them to guide the separation and utilization of macerals in a direction that is both economically viable and environmentally sustainable, thus paving the way for future industrial implementation.

## 5. Conclusions

YLV with a vitrinite content of 80.80% is obtained through a stepwise crushing method, and YLI with an inertinite content of 83.08% is obtained through a primary crushing method. These processes can improve the separation degree of the screening method, which does not require water and chemical reagents.

YLV exhibits the highest content of highly reactive functional groups, featuring a relative aliphatic carbon content of 35.88%, as well as the longest aliphatic and bridging chains. These structural characteristics collectively contribute to a higher oil yield of YLV compared to YLC and YLI. In contrast, YLI exhibits an aromatic carbon relative content of 66.60%, accompanied by the highest degree of aromatic condensation. These structural characteristics collectively lead to a lower oil yield than YLC and YLV. Due to significant structural differences among various macerals, there is a strong correlation between the structure of each maceral and the composition of its liquefaction oil. These findings provide new insights for the high-value utilization of coal and the production of aromatic-rich oil.

Furthermore, the findings of this study provide partial foundational data to support the LCA and TEA. In future work, LCA and TEA should be employed to evaluate the environmental sustainability and economic feasibility.

## Figures and Tables

**Table 1:** Maceral composition of YLC.

Sample	Maceral Content (V%)			
	Exinite	Vitrinite	Inertinite	Mineral
YLC	0.90	52.20	46.90	0.00

**Table 2:** Maceral composition of Yili coal with different particle sizes.

Particle Sizes (mm)	Maceral Content (V%)		
	Exinite	Vitrinite	Inertinite
>0.5	1.20	61.00	37.80
0.5–0.25	0.88	47.56	51.56
0.25–0.125	0.85	38.38	60.77
0.125–0.074	0.59	30.87	68.54
0.074–0.045	0.23	24.83	74.94
<0.045	0.19	16.73	83.08

**Table 3:** Maceral composition of Yili coal with different grinding times.

Times	Particle Sizes (mm)	Maceral Content (V%)		
		Exinite	Vitrinite	Inertinite
1	>0.074	1.00	53.60	45.40
2	>0.074	1.23	56.91	41.86
3	>0.074	1.27	64.25	34.48
4	>0.074	1.38	73.39	25.23
5	>0.045	1.40	80.80	17.80

**Table 4:** Proximate and ultimate analyses of different coal samples.

Coal Samples	Proximate Analysis (wt.%)			Ultimate Analysis (daf, wt.%)				
	M_ad_	A_d_	V_daf_	C	H	N	S	O ^a^
YLC	1.13	3.78	31.82	77.43	4.12	1.13	0.17	17.15
YLV	1.23	3.61	32.23	76.45	4.18	1.15	0.26	17.96
YLI	0.80	4.01	31.20	77.14	4.06	1.14	0.14	17.52

a: Obtained by the method of subtraction.

**Table 5:** Peak fitting of coal samples at 3000–2800 cm^−1^ and 1800–1000 cm^−1^.

Peak Number	Peak Center (cm^−1^)	Functional Groups	Area Percentage (%)		
			YLC	YLV	YLI
1	2947	ν_as_CH_3_- (aliphatic)	22.48	20.72	23.62
2	2921	ν_as_CH_2_- (aliphatic)	25.16	26.42	24.36
3	2897	νR_3_CH (aliphatic)	23.47	24.58	22.82
4	2873	ν_s_CH_3_- (aliphatic)	6.03	4.48	6.91
5	2849	ν_s_CH_2_- (aliphatic)	22.86	23.80	22.29
6	1713	ν_C=O_ (carboxylic acid)	3.20	2.94	3.29
7	1670	ν_C=O_	8.00	7.87	8.16
8	1587	ν_C=C_ (aromatic)	32.82	32.37	33.34
9	1442	δ_as_CH_2_, CH_3_	15.84	16.17	15.46
10	1373	δ_s_CH_2_, CH_3_	7.71	8.05	7.37
11	1314	ν_C-O_ (phenolic)	7.76	7.95	7.43
12	1254	ν_C-O_ (phenolic)	10.53	10.68	10.40
13	1187	ν_C-O_ (alcohol)	8.74	9.14	8.42
14	1114	ν_C-O_ (aromatic ethers)	4.11	3.84	4.51
15	1039	ν_C-O-C_ (alkyl ethers)	1.29	0.99	1.62

**Table 6:** Semi-quantitative ratios derived from FTIR spectra.

Semi-Quantitative Index	Calculated Value		
	YLC	YLV	YLI
Aliphatic hydrocarbon content	4.61	5.57	3.50
Chain length	1.68	1.99	1.53
Relative aliphatic/aromatic ratio	0.48	0.50	0.46

Aliphatic hydrocarbon content: (CH_2_ + CH_3_)/C=C = (A_2947_ + A_2921_ + A_2873_ + A_2849_)/A_1587_ × 100. Chain length: CH_2_/CH_3_ = (A_2921_ + A_2849_)/(A_2947_ + A_2873_). Relative aliphatic/aromatic ratio: (asymmetric CH_2_, CH_3_)/C=C = A_1442_/A_1587_.

**Table 7:** The Raman peak fitting parameters.

Samples	D_1_ (%)	D_3_ (%)	D_4_ (%)	G (%)	D_1_/G	D_3_/G	D_4_/G	D_all_/G
YLC	31.24	28.07	18.90	21.79	1.43	1.29	0.87	3.59
YLV	28.71	29.64	21.01	20.64	1.39	1.44	1.02	3.84
YLI	33.14	26.92	17.41	22.53	1.47	1.19	0.77	3.44

**Table 8:** Carbon structural parameters of coal.

Peak Number	Assignments	Symbols	Chemical Shift (ppm)	Area Percentage (%)		
				YLC	YLV	YLI
1	R-CH_3_	f_al_^1^	12.93	2.88	2.74	3.06
2	Ar-CH_3_	f_al_^a^	19.27	3.50	3.14	3.70
3	RCH_2_CH_3_	f_al_^2^	23.87	2.23	2.97	1.72
4	-CH_2_-CH_2_-CH_2_-	f_al_^3^	29.80	8.72	10.68	6.17
5	-CH-, -C-	f_al_^4^	38.70	8.71	10.32	7.83
6	CH_3_OCH_2_-	f_al_^O1^	53.03	2.30	2.01	2.47
7	-CH_2_OCH_2_-	f_al_^O2^	74.67	0.99	0.84	1.13
8	RCH_2_OH, **>**CHOH	f_al_^O3^	89.20	2.61	3.18	2.05
	Aliphatic	f_al_		31.94	35.88	28.13
9	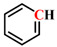	f_ar_^H^	119.67	28.89	27.64	29.16
10	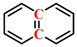	f_ar_^B^	128.27	16.10	14.77	17.56
11	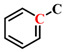	f_ar_^S^	138.50	9.30	9.63	9.03
12	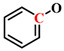	f_ar_^O^	152.83	9.67	8.72	10.85
	Aromatic	f_ar_		63.96	60.76	66.60
13	-COOH, -COOR	f_a_^C1^	174.67	3.07	2.47	3.79
14	**>**C=O, -CHO	f_a_^C2^	200.00	1.03	0.89	1.48
	C=O	f_a_^C^		4.10	3.36	5.27

The red “C” in Table 8 corresponds to the carbon types of the peak numbers in Figure 3.

**Table 9:** Structural parameters of YLC, YLV and YLI.

Structure Parameters	Samples		
	YLC	YLV	YLI
C_n_	2.64	2.94	2.07
C_b_	2.78	3.57	2.09
σ_-C_ (%)	14.54	15.84	13.56
σ_-O_ (%)	15.12	14.35	16.29
X_b_	0.25	0.24	0.26

C_n_: Average length of methylene chain, C_n_ = 7/5 (f_al_^2^ + f_al_^3^)/(f_ar_^S^ − f_al_^a^). C_b_: Average length of bridge chain, C_b_ = 2 (f_al_^3^ + f_al_^4^ + f_al_^O2^)/(f_ar_^S^ − f_al_^a^ − f_al_^2^ + f_ar_^O^). σ_-C_: -C aromatic ring substitution degree, σ_-C_ = (f_ar_^S^)/f_ar_. σ_-O_: -O aromatic ring substitution degree, σ_-O_ = (f_ar_^O^)/f_ar_. X_b_: Bridgehead carbon quantity, X_b_ = f_ar_^B^/f_ar_.

## Data Availability

Data will be made available on request.

## Abbreviations

The following abbreviations are used in this manuscript:

YLC	Yili coal
YLI	Inertinite-rich concentrates of Yili coal
YLV	Vitrinite-rich concentrates of Yili coal
DCL	Direct coal liquefaction
Oil_YLC_	The liquefied oils of YLC
Oil_YLV_	The liquefied oils of YLV
Oil_YLI_	The liquefied oils of YLI
THN	Tetralin
